# Nanoporous Silica Aerogels Derived from Tailings via Alkali Fusion Activation and Resin-Free Purification

**DOI:** 10.3390/ma19214514

**Published:** 2026-10-27

**Authors:** Kang Ma, Qianqian Wang, Xiaodong Shen

**Affiliations:** 1 College of Materials Science and Engineering, Nanjing Tech University, Nanjing 211816, China; ma411422@163.com; 2 State Key Laboratory of Materials-Oriented Chemical Engineering, Nanjing 211816, China

**Keywords:** copper–nickel tailings, silica aerogel, thermal conductivity insulation, ultrasonic purification

## Abstract

This study presents a novel method for synthesizing nanoporous silica aerogels from copper–nickel tailings (CTs) via ambient pressure drying using a resin-free purification approach. CTs are an underutilized industrial solid waste rich in silicates, typically landfilled rather than valorized. To address the high raw material costs and complexity of supercritical drying in conventional silica aerogel synthesis, a potentially advantageous method using CT-derived silicon was developed. Box–Behnken design (BBD) was used to guide the alkali-melting and acid-leaching processes. An intermittent ultrasonic washing method instead of resin-free purification realized the removal of most impurity ions as well as optimized the pore structures of the aerogels. Under this technological condition, a silica aerogel with a specific surface area of 738.92 m^2^/g and a thermal conductivity of 0.03571 W/(m·K) was obtained, showing that the method can lower the production cost of silica aerogel by over 20% compared to organic silicon sources, and with less new solid-waste generation during the process. This work provides a potential and feasible method for transforming silicate-rich industrial waste into high-value-added silica aerogel materials, compliant with the development of the circular economy.

## 1. Introduction

Over the past few decades, the mining industry has developed rapidly, resulting in 65–80 billion tons of mining wastes accumulating annually, of which tailings constitute the largest fraction, reaching 10–15 billion tons per year [1]. Their continuous accumulation imposes severe environmental pressure, posing a major obstacle to the sustainable development of mining regions. Tailings are always used for mine backfilling at the largest scale [2,3], which leads to the infiltration of harmful substances into the ground, land pollution and vegetation degradation near the mine. What is more serious is that the strength of fine tailings after backfilling is insufficient, and the mining area collapses, which seriously endangers life and safety [4]. Therefore, the treatment and utilization of tailings are urgently needed. CTs and iron tailings hold significant potential for producing low-cost silica aerogels (SAs), which are rich in SiO_2_ and silicate minerals with high dissolution possibilities [5,6].

SAs are nanoporous, three-dimensional, structured materials characterized by a high specific surface area, porosity, low density, and thermal conductivity [7,8,9,10,11,12]. These outstanding properties have enabled their wide application in thermal insulation [13], catalysis [14], acoustics [15], and toxic [16]/organic pollutant treatment [17]. In particular, the increasing demand for insulation in China’s fourth-generation residences positions SA as a promising material for future large-scale application [18].

However, high-quality SAs are typically synthesized with organosilicon precursors (e.g., Tetraethoxysilane (TEOS) [19], (3-Mercaptopropyl) trimethoxysilane (MPTMS) [20], Methyltrimethoxysilane (MTMS) [21]) and dried using the supercritical method, which inevitably raises production costs and restricts their large-scale application in the construction industry. To decrease the cost of SAs, raw material replacement and drying technology could be the most probable way [22]. Researchers have devoted efforts to synthesizing SAs with diverse solid wastes, such as fly ash, coal gangue, waste glass and gold tailings [23,24,25,26,27]. Among the diverse preparation processes, the alkali fusion–acid-leaching–sol–gel method [28] is the most prevalent, followed by the direct conversion method, one-pot method [29], hydrothermal-assisted method [18] and composite reinforcement method [30]. In terms of drying regimes, supercritical CO_2_ drying and freeze drying are usually adopted to retain the intact pore structure and monolithic morphology [30]. Atmospheric pressure drying is developing as a low-cost and scalable technique [31].

Nevertheless, the large-scale application and industrialization of solid-waste-derived aerogels still face prominent technical and economic bottlenecks. Firstly, low silicon leaching efficiency was always the bottleneck during previous research [32]. Previous studies demonstrated the poor dissolution performance of olivine and silicate-rich slags/tailings, accompanied by low silicate concentrations in both organic and inorganic acidic solutions [33,34]. Alkali fusion has proven to be an effective strategy to activate highly crystalline silicates, which converts inert crystalline silicates into sodium silicates and thereby facilitates silicon extraction in water solutions [35]. Secondly, fluctuations in the chemical composition of industrial solid wastes and complex impurity ions readily induce gel network heterogeneity and pore structure deterioration, leading to the unstable performance of aerogels [36]. Meanwhile, in the process of atmospheric drying, insufficient solvent replacement easily causes the irreversible shrinkage of wet gel [31], which consequently causes pore collapse and the degradation of the thermal insulation performance. In addition, the utilization of solid wastes as a silica source for aerogel synthesis should fulfill the principles of the circular economy, which are “reduce, reuse, and recycle”, in the processes of SA production and consumption. Therefore, there are still obstacles for the synthesis of SA with solid wastes with good thermal insulation performance and low cost [37,38].

To address the above issues, SA materials were prepared in this study via ambient pressure drying using massively stockpiled CTs as raw materials. From the perspective of the circular economy, CTs are stockpiled mine tailings. Fabricating aerogels with CTs as the silicon source mitigates the environmental and soil hazards caused by waste accumulation and reduces reliance on commercial high-purity silicon sources such as TEOS and TMOS. The alkali fusion–acid-leaching processes are adopted to selectively dissolve silicate minerals in tailings, yielding silicon-containing leachate for the subsequent sol–gel reaction. The Box–Behnken design (BBD) [39] is used to investigate the interactive effects of multiple independent variables, including the alkali fusion temperature, alkali-to-solid mass ratio and acid-to-solid mass ratio, on the response values (reaction degree and gelation time). The trends of the reaction degree and gelation time with respect to the alkali fusion temperature, alkali-to-solid mass ratio and acid-to-solid mass ratio can be deduced from the BBD analysis, which could provide insights for subsequent optimization attempts. A resin-free purification strategy is applied prior to hydrophobic modification, in which impurities are removed by water washing and solvent displacement. This eliminates the ion-exchange operation and avoids waste liquid generated from resin regeneration. Therefore, the utilization of CTs as a silica source for aerogel synthesis aligns well with circular economy principles. Finally, the temperature resistance and thermal insulation performances of the as-prepared aerogel particles are characterized. This work provides an efficient route for converting silicate-rich tailings into high-value aerogel materials.

## 2. Materials and Methods

### 2.1. Preparation of Experimental Materials

**Figure 1:**
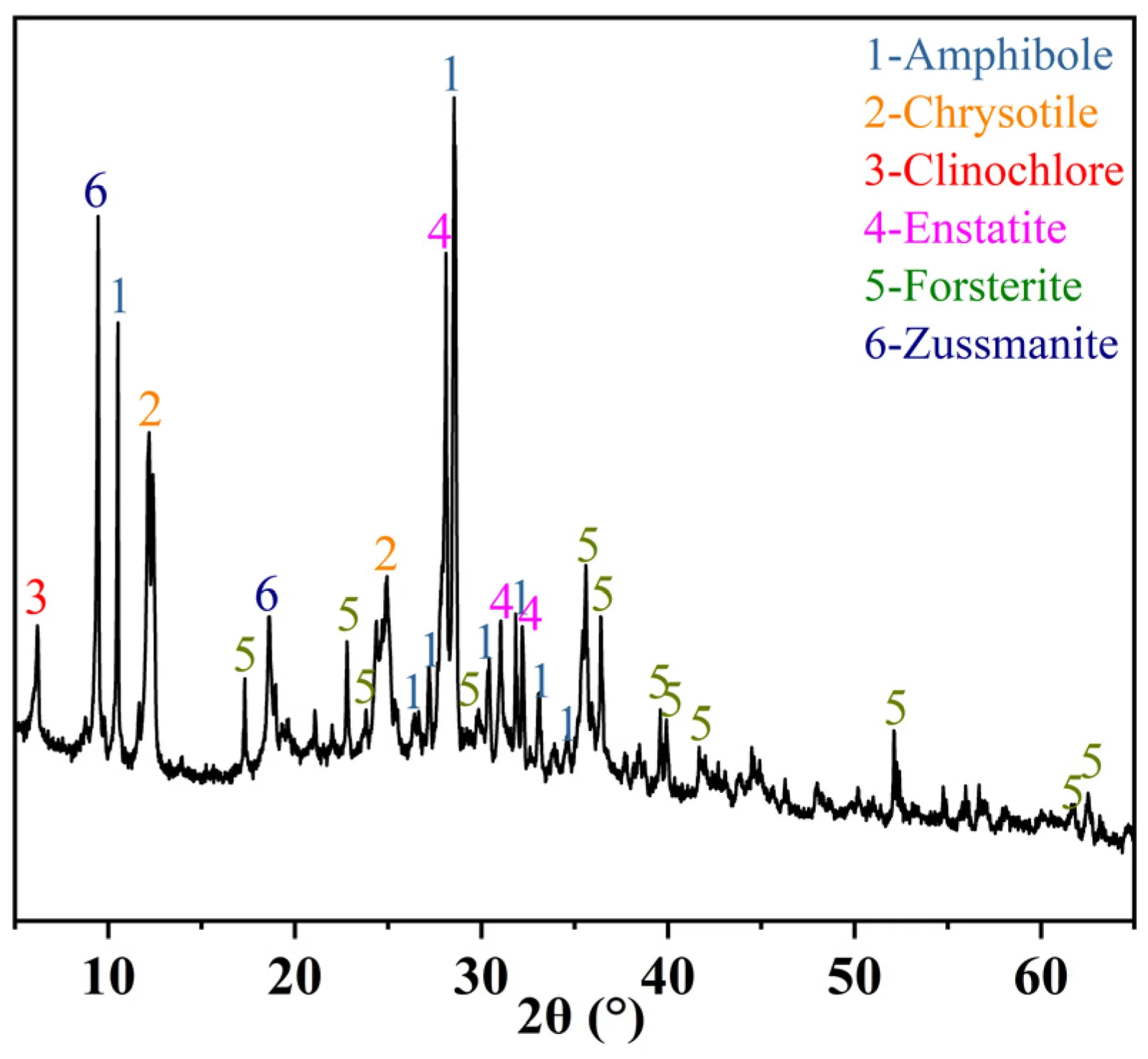
XRD pattern of CTs.

The CTs were obtained from Kalatongke, Xinjiang Province, ground with an SM-500 ball mill (Wuxi Jianyi Instrument & Machinery Co, Wuxi, Jiangsu, China) for 30 min, and sieved through a 75 μm square-hole sieve for subsequent tests. X-ray diffraction (XRD) (Rigaku MiniFlex 600 benchtop powder X-ray diffractometer, Tokyo, Japan) (Figure 1) identified the main crystalline minerals of the CT powder as forsterite, enstatite, amphibole, chrysotile, and zussmanite. X-ray fluorescence (XRF) (Skyray Instrument, Kunshan, China) (Table 1) showed that SiO_2_ accounted for 40.40 wt.% of the total chemical composition. Experimental reagents are listed in Table 2. The abbreviations used in this article are listed in Table 3.

### 2.2. Aerogel Synthesis Process

**Figure 2:**
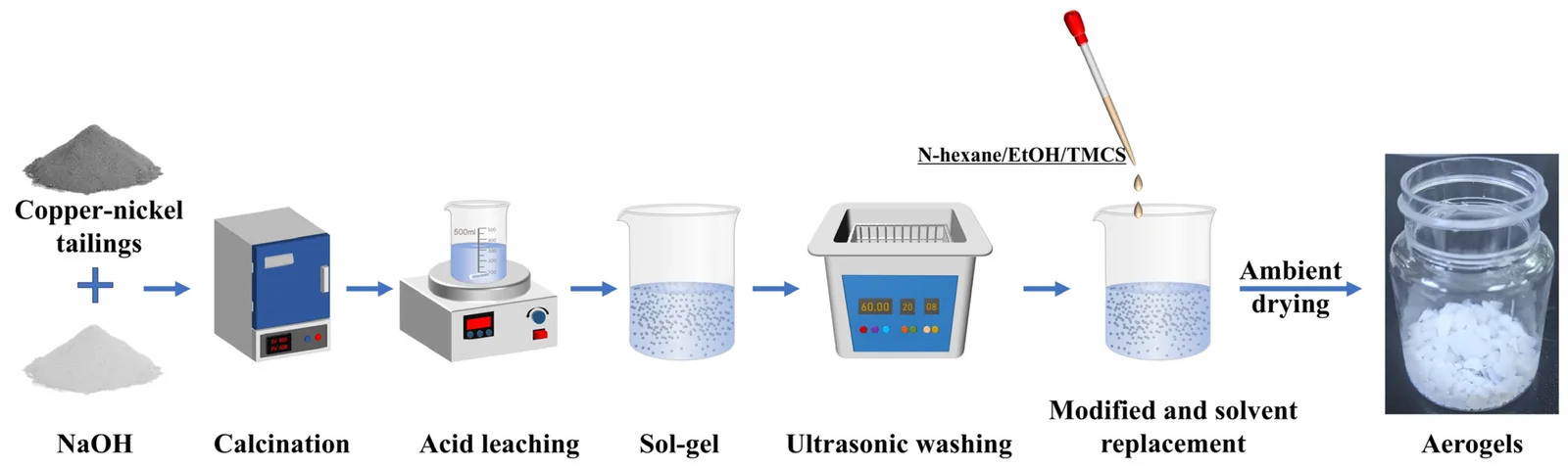
The preparation process and schematic diagram of SA with CTs as the silicon source.

The specific steps for preparing SiO_2_ aerogel from CTs under APD were as follows, and the flow chart is shown in Figure 2.

Firstly, the as-sieved CT samples and NaOH were ground in an agate mortar for 10 min to mix them evenly and were then calcined at different temperatures. Then, the calcined product was ground into fine powder and slowly poured into a beaker containing pre-prepared 5 mol/L HNO_3_ solution at room temperature, followed by magnetic stirring for 30 min. Afterwards, the solutions were separated with a centrifuge for 2 min at 8000 rpm. The supernatants are silica sol and can form gels at room temperature quickly.

To stabilize the gel network structure, the gels were aged for 12 h at room temperature. Then, normal washing and ultrasonic washing were combined to remove the impurity ions in the gel network. The wet gels were immersed in deionized water at a volume ratio of 4:1 (deionized water:wet gel). The wet gel was washed 4 times with an interval of 6 h. To enhance the removal efficiency of impurity ions, the samples were sonicated in an ultrasonic cleaner for 10 min following each deionized water replacement.

After washing, wet gels were immersed in EtOH at a volume ratio of 4:1 (EtOH: wet gel). The EtOH was replaced 4 times with an interval of 6 h each time, resulting in the formation of alcogel. Hydrophobic modification on alcogels had to be conducted by immersing them into a mixed solution of n-hexane:EtOH:Trimethylchlorosilane (TMCS) at a volume ratio of 4:1:1. During this process, solvent substitution happened, which is very important for the lateral APD process. Solvent substitution was conducted two times with an interval of 12 h, and the volume ratio of the mixed solution to the alcogels was 4:1.

Finally, these wet gels were placed in an air blast drying oven at 60 °C for 4 h and the SA was obtained.

### 2.3. Experimental Method Based on BBD

The reaction degree of CTs, i.e., the utilization degree of CTs to produce silica sol, is very import to the SA synthesis, since the disposition of residues should be considered. The alkaline fusion and acid-leaching processes are the key to improve the reaction degree of CTs as well as the gelation time of silica sol. The reaction degree of CTs through the alkali fusion–acid-leaching process is defined in Equation (1) [32]:

(1) $$\mathrm{Reaction}\;\mathrm{degree}\;\left(CTs\right)=\left[1\;-\;\frac{m\prime }{m\;\times \;(1\;-\;p)}\right]\;\times \;100\%$$

where m represents the original mass of CTs; m′ represents the mass of filtered residues after the alkali fusion and acid-leaching processes; p represents the loss on ignition of raw CTs based on XRF.

BBD was used to investigate the effects trend of the alkali fusion and acid-leaching parameters on the reaction degree of CTs and the gelation time of silica sol. Design expert software (DX10, Stat-Ease, Inc., Minneapolis, MN, USA) was used for the BBD and for subsequently fitting the experimental data.

According to the experimental design scheme of the Box–Behnken response surface, the experimental results can be fitted to build up the relationships between the response and different influence factors. The general form of the model is:

(2) $$Y=\beta_{0}+\sum_{i=1}^{k}\beta_{i}X_{i}+\sum_{i=1}^{k}\beta_{ii}X_{i}^{2}+\sum_{i<j}\beta_{ij}X_{i}X_{j}+\varepsilon$$

where Y is the predicted response; $\beta_{0}$ is the constant term; $\beta_{i}$, $\beta_{ii}$ and $\beta_{ij}$ represent the linear, quadratic and interaction coefficients, respectively; $X_{i}$ and $X_{j}$ are the coded independent variables; ε is the residual error.

The gelation time was recorded from solid–liquid separation to the time when the solutions could not flow. The following parameters were used to assess the reliability of the quadratic regression model established by BBD: the coefficient of determination (R^2^), adjusted coefficient of determination (Adj R^2^), and coefficient of variation (CV%). The data were interpreted in the fitting process. The R^2^ value represents the fraction of total response variance explained by the model, while Adj R^2^ adjusts the R^2^ by accounting for the number of model terms. CV% reflects the relative experimental error; a higher CV% indicates the poorer repeatability of measurements.

The experimental design process took the calcination temperature, alkali-to-solid mass ratio and acid-to-solid mass ratio as influencing factors (A, B, C) and the reaction degree (Y_1_) and initial gelation time (Y_2_) as response values. The model was used to visualize the variation trends between variables and response values. The factors and horizontal parameters used in this experiment are shown in Table 4. The calcination temperature was set between 600 and 800 °C, the alkali-to-solid mass ratio was established at 0.5 to 1, and the acid-to-solid mass ratio was set at 6 to 10. The experimental design results of the response surface are shown in Table 5, with 13~17 as the central experimental points for estimating the experimental error.

### 2.4. Characterization Methods

#### 2.4.1. X-Ray Diffraction

An X-ray diffractometer (Rigaku MiniFlex 600 benchtop powder X-ray diffractometer, Rigaku Corporation, Akishima, Tokyo, Japan) was used for the XRD analysis of raw materials and alkali fusion-treated samples. The X-ray tube was operated at 40 kV and 15 mA. Scanning was performed from 5–65° at a scan rate of 10°/min and a step size of 0.02°.

#### 2.4.2. Contact Angle

The hydrophobicity of the SA was characterized using a DY10-1C optical contact angle goniometer (Shanghai Zhongchen Digital Technology Equipment Co., Ltd., Shanghai, China). The sample was ground and sieved through a 75 μm square sieve. The aerogel powder was uniformly spread on a glass slide, and water droplets were deposited onto the powder surface. The contact angle was calculated from captured images. The instrument operated at an input voltage of 200 V, an output voltage of 12 V and a power of 10 W.

#### 2.4.3. Specific Surface Area Measurement

A BELSORP MINI X fully automated specific surface area and pore size analyzer (MicrotracBEL, Osaka, Japan) was adopted to characterize the specific surface area and pore structure of the SA samples. Prior to measurement, the SA aerogels were vacuum-degassed at 120 °C for 12 h to remove adsorbed volatile contaminants. Nitrogen adsorption isotherms were acquired at 77 K across a series of preset pressures. The specific surface area, average pore size and pore size distribution were calculated from the adsorption isotherms.

#### 2.4.4. Thermal Conductivity Measurement

The thermal conductivity of the aerogel samples was measured using a Hot Disk TPS 2500S Thermal Constants Analyzer (Hot Disk AB, Gothenburg, Sweden) based on the transient plane source method. Two aerogel slabs of identical dimensions were clamped with the Hot Disk probe sandwiched between them. Appropriate pressure was applied via a pressure screw. Parameters, including the transient temperature rise, characteristic time and residual, were adjusted until stable thermal conductivity data were obtained at 30 °C. The instrument thermal conductivity range was 0.01–100 W/(m·K).

#### 2.4.5. Thermogravimetry–Differential Scanning Calorimetry (TG-DSC)

TG-DSC tests were performed on a Netzsch STA 449 F5 Jupiter^®^ simultaneous thermal analyzer (NETZSCH-Gerätebau GmbH, Selb, Germany) to record the weight loss and thermal events of the aerogels during heating. Samples were heated from room temperature to 1000 °C at 20 °C/min under an N_2_ atmosphere with a gas flow rate of 50 mL/min. The thermal resistance of the aerogels was evaluated from the TG-DSC curves.

#### 2.4.6. Scanning Electron Microscope (SEM)

A scanning electron microscope (ZEISS Gemini SEM 360 field emission scanning electron microscope (FE-SEM), Carl Zeiss Microscopy GmbH, Jena, Germany) was used to observe the micro-morphology of the samples at an accelerated voltage of 10 kV. Due to the low conductivity of the samples, the samples were glued on the sample table with conductive adhesive and then gold-sprayed before the test.

#### 2.4.7. Thermal Stability Evaluation

A SX-4-10 box-type resistance muffle furnace (Tianjin Taisite Instrument Co., Ltd., Tianjin, China) was used for the high-temperature calcination of the aerogel powders. The aerogel powders were sieved through a 75 μm square-hole sieve before calcination. Samples were heated at 10 °C/min and held at target temperatures (300 °C, 400 °C, 450 °C, 500 °C, 550 °C) for 0.5 h.

#### 2.4.8. Fourier Transform Infrared Spectroscopy (FTIR)

An IR Spirit-X Fourier transform infrared spectrophotometer (Shimadzu Corporation, Kyoto, Japan) was used to characterize sample functional groups. The sample was mixed with dried KBr powder, ground into fine powder and pressed into a transparent pellet. Spectra were collected in the wavenumber range of 4000–400 cm^−1^ with a resolution of 4 cm^−1^. Background scanning was carried out before sample measurement.

## 3. Results and Discussion

### 3.1. Influence Factors on Reaction Degree of CTs

Design expert software was used to fit the experimental data, and the regression equation was used for the reaction degree (Y_1_) as a function of the calcination temperature (A), alkali-to-solid mass ratio (B) and acid-to-solid mass ratio (C). The reaction degrees of the CTs were determined according to Equation (1) and are listed in Table 4. The analysis of the variance in the reaction degree is presented in Table 6. The model produced a correlation coefficient (R^2^) of 0.8619. The regression model follows Equation (3) (the insignificant factors were deleted in the fitting process):

(3) $$Y_{1}\;=\;76.51\;+\;8.22A\;+\;3.53C\;-\;4.90A^{2}$$

The R^2^ is high; however, the lack-of-fit parameter is significant. Therefore, the regression model could only be used to show the trend of the parameter change for the aerogel synthesis. It could not be used as an optimization model.

**Figure 3:**
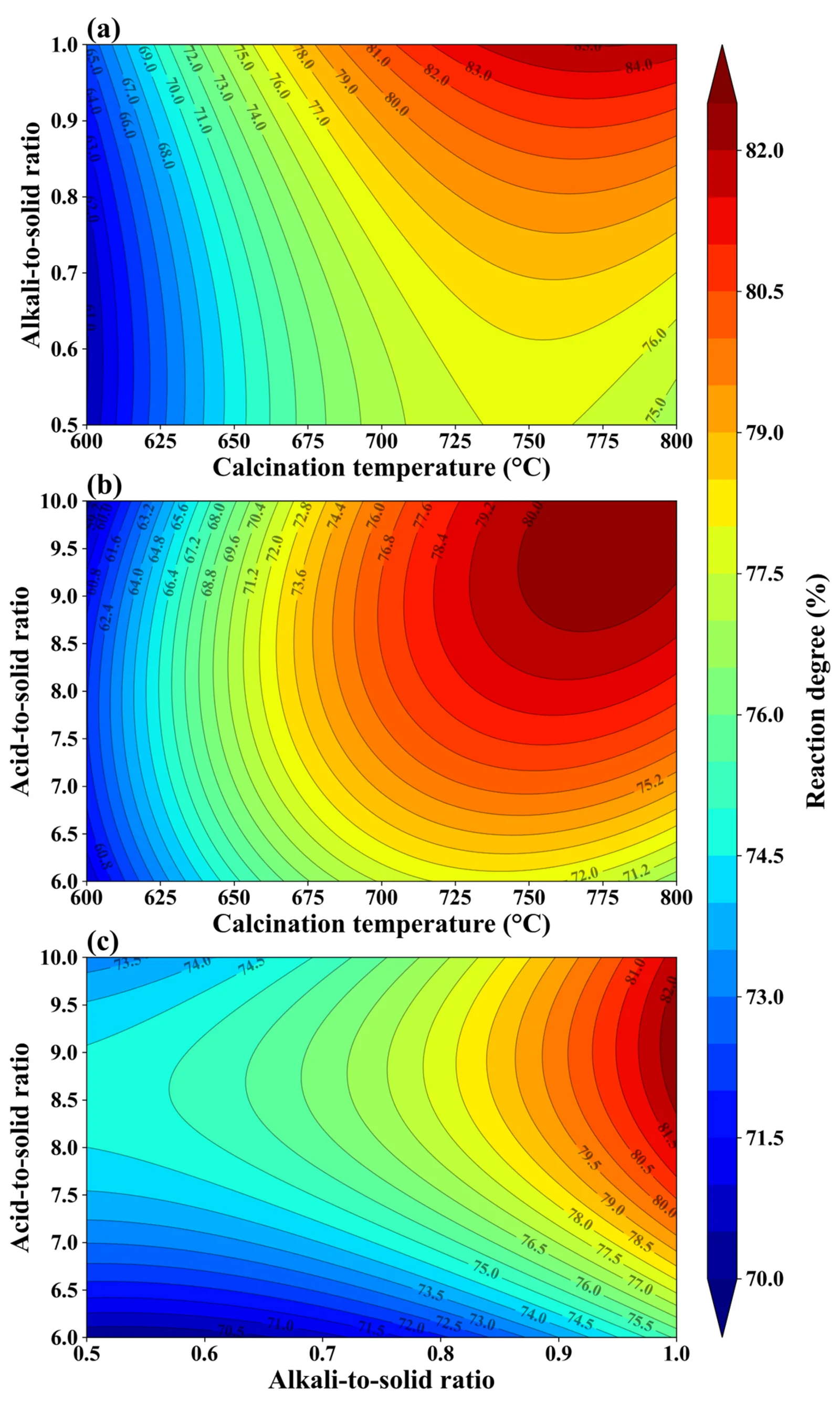
Contour plot of reaction degrees of CTs under different correlation influence factors: (**a**) calcination temperature and alkali-to-solid mass ratio; (**b**) calcination temperature and acid-to-solid mass ratio; (**c**) alkali-to-solid mass ratio and acid-to-solid mass ratio.

As shown in Figure 3a, the steepest descent path is mostly along the calcination temperature, indicating that it has a higher influence on the reaction degree than the alkali–solid mass ratio. In addition, when the calcination temperature was in the range of 600 and 750 °C, the increase in the alkali-to-solid mass ratio (B) was negatively correlated with the reaction degree. When the calcination temperature was higher than 750 °C, increasing the alkali-to-solid mass ratio was beneficial for the reaction degree. From Figure 3b, the steepest descent path is mostly along the calcination temperature as well. When the calcination temperature was higher than 750 °C, increasing the acid-to-solid mass ratio obviously improved the reaction degree. From Figure 3c, the steepest descent path is mostly along the acid-to-solid mass ratio, indicating that the acid-to-solid mass ratio has a greater influence on the reaction degree than the alkali-to-solid mass ratio. In the region of the acid-to-solid mass ratio higher than 7 and of the alkali-to-solid mass ratio higher than 0.64, the reaction degree can be improved faster with the increase in the acid-to-solid mass ratio, certifying that the acid-to-solid mass ratio is more important for the reaction degree than the alkali-to-solid mass ratio.

Therefore, it can be concluded that the influence factors on the reaction degree of CTs were ordered in the following sequence: calcination temperature > acid-to-solid mass ratio > alkali-to-solid mass ratio.

**Figure 4:**
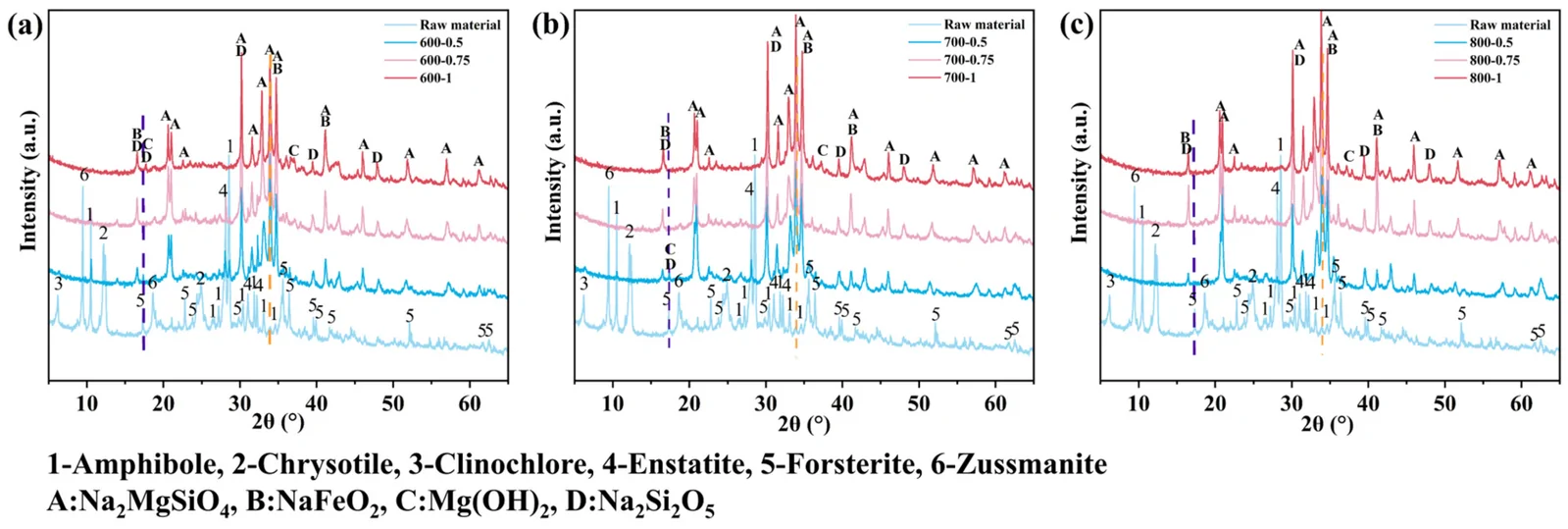
XRD patterns of alkali fusion products at different temperatures: (**a**) 600 °C, (**b**) 700 °C and (**c**) 800 °C (The purple dashed lines denote the forsterite mineral phase peaks that disappear after alkali fusion, and the orange dashed lines represent the mineral phase peaks of soluble sodium salts formed after alkali fusion.).

Raw CTs mainly contain silicate and aluminosilicate minerals according to the XRD results in Figure 1, and adding alkali was an effective method to activate CTs. XRD was used to determine the phase change during the alkali fusion process with different alkali–solid ratios and calcination temperatures. The results in Figure 4 show that the diffraction peaks of calcium and magnesium silicate minerals, such as olivine and amphibole, weakened or disappeared after the alkali fusion reaction. After alkali fusion, Na_2_MgSiO_4_ and Na_2_Si_2_O_5_ were examined from the XRD patterns in Figure 4. According to the XRD results in Figure 4 and Reference [40], chemical reactions during the alkali fusion process can be summarized as Equations (4)–(7):

(4) $${2Mg}_{2}{SiO}_{4}+6NaOH={Na}_{2}{MgSiO}_{4}+3{Mg(OH)}_{2}{+Na}_{4}{SiO}_{4}$$

(5) $${Na}_{2}{MgSiO}_{4}+2NaOH={Mg(OH)}_{2}{+Na}_{4}{SiO}_{4}$$

(6) $$2{\left(Mg,Fe\right)}_{2}{SiO}_{4}+8NaOH+3O_{2}=4{NaFeO}_{2}{+4Mg(OH)}_{2}+{2Na}_{2}{SiO}_{3}$$

(7) $$2{Na}_{4}{SiO}_{4}={Na}_{2}{{Si}_{2}O_{5}+3Na}_{2}O$$

As shown in Figure 4a, the diffraction peak of forsterite still exists in the orange dotted box, indicating that 600 °C is not high enough to fully transform the calcium and magnesium silicate minerals into Na_2_MgSiO_4_ and Na_2_Si_2_O_5_. As the temperature rises, the solid-state reaction accelerates, leading to the conversion of more calcium and magnesium silicate minerals into Na_2_MgSiO_4_ and Na_2_Si_2_O_5_. In Figure 4b,c, the diffraction peak intensity of the product Na_2_MgSiO_4_ marked with the green dotted line is obviously higher than that in Figure 4a. Therefore, the recommended temperature should be higher than 700 °C, aiming to improve the silicate transformation rate, which is a key point to improve the reaction degree of CTs for the synthesis of silica sol.

### 3.2. Influence Factors on Gelation Time of Silica Sol

The gelation time was defined as the time elapsed from the completion of sol preparation to the point at which the silica sol lost macroscopic fluidity and ceased to flow within 30 s when the beaker was tilted to 45°. The gelation time results are listed in Table 5. The quadratic model built on raw Y_2_ exhibited a high CV due to heteroscedasticity. Natural logarithmic transformation (lnY_2_) was applied to stabilize the variance in residuals. Design expert software was used to fit the experimental data and the regression equation for the gelation time (Y_2_) as a function of the calcination temperature (A), alkali-to-solid mass ratio (B) and acid-to-solid mass ratio (C). The analysis of the variance in the gelation time is listed in Table 7. The model produced a correlation coefficient (R^2^) of 0.9126. The regression function was as Equation (8):

(8) $$LnY_{2}\;=\;5.92\;-\;0.38A\;+\;0.78B\;-\;0.65C$$

The R^2^ is high; however, the lack-of-fit parameter is significant. Again, the regression model could only be used to show the trend of the parameter change for the aerogel synthesis.

**Figure 5:**
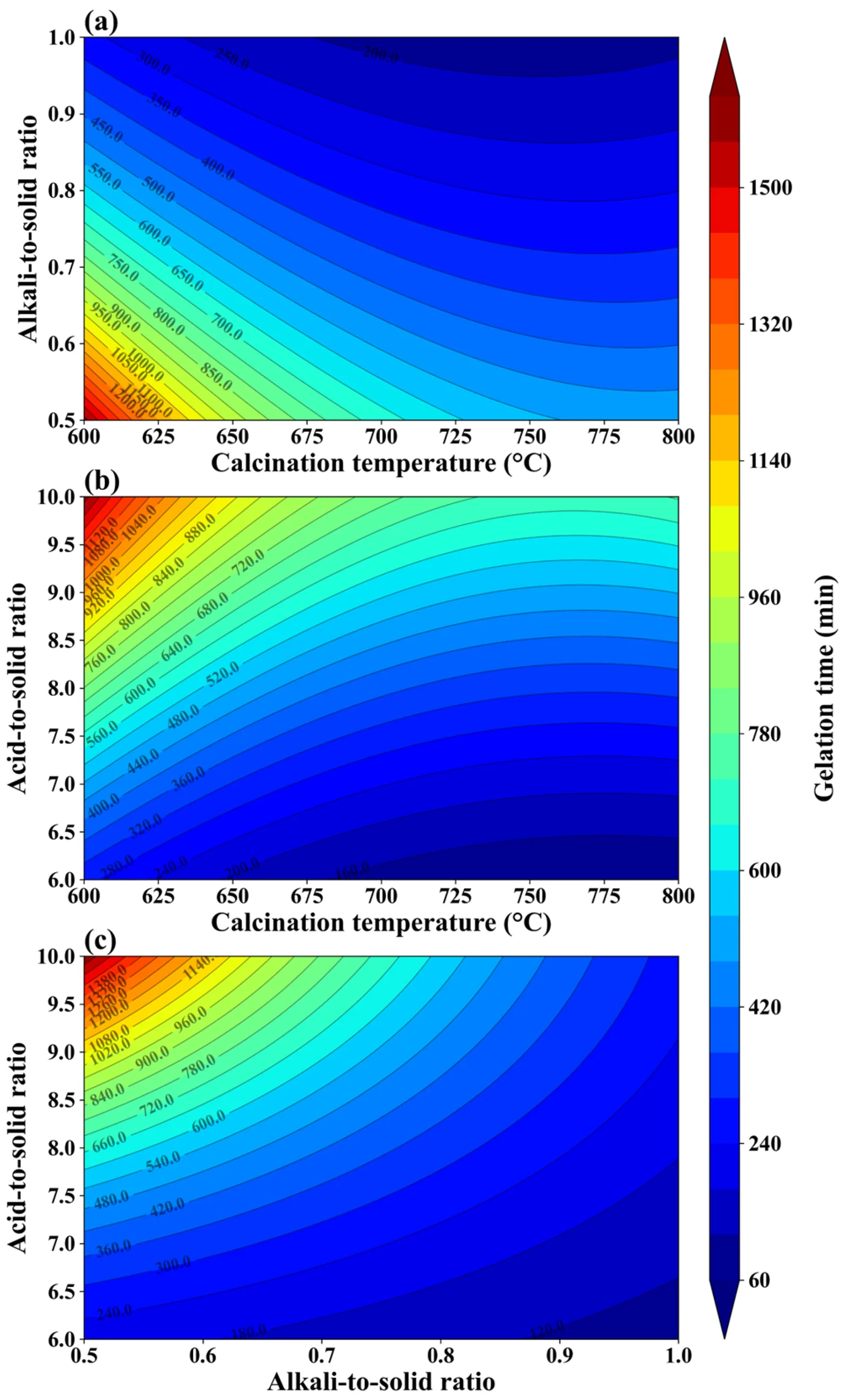
Contour plot of gelation time of silica sol under different correlation influence factors: (**a**) calcination temperature and alkali-to-solid mass ratio; (**b**) calcination temperature and acid-to-solid mass ratio; (**c**) alkali-to-solid mass ratio and acid-to-solid mass ratio.

As shown in Figure 5, the factors with the most influence on the gelation time of silica sol are the acid-to-solid mass ratio and alkali-to-solid mass ratio. The calcination temperature is not a significant parameter.

Therefore, based on the above BBD analysis results and experimental work, we tried different process parameters, such as different calcination temperatures (700 °C, 800 °C), alkali-to-solid mass ratios (0.75, 0.875) and acid-to-solid mass ratios (8, 10), for further synthesis and performance characterization.

### 3.3. Silica Aerogel Characterization and Performance

#### 3.3.1. Pore Structure and Specific Surface Area Analysis

**Figure 6:**
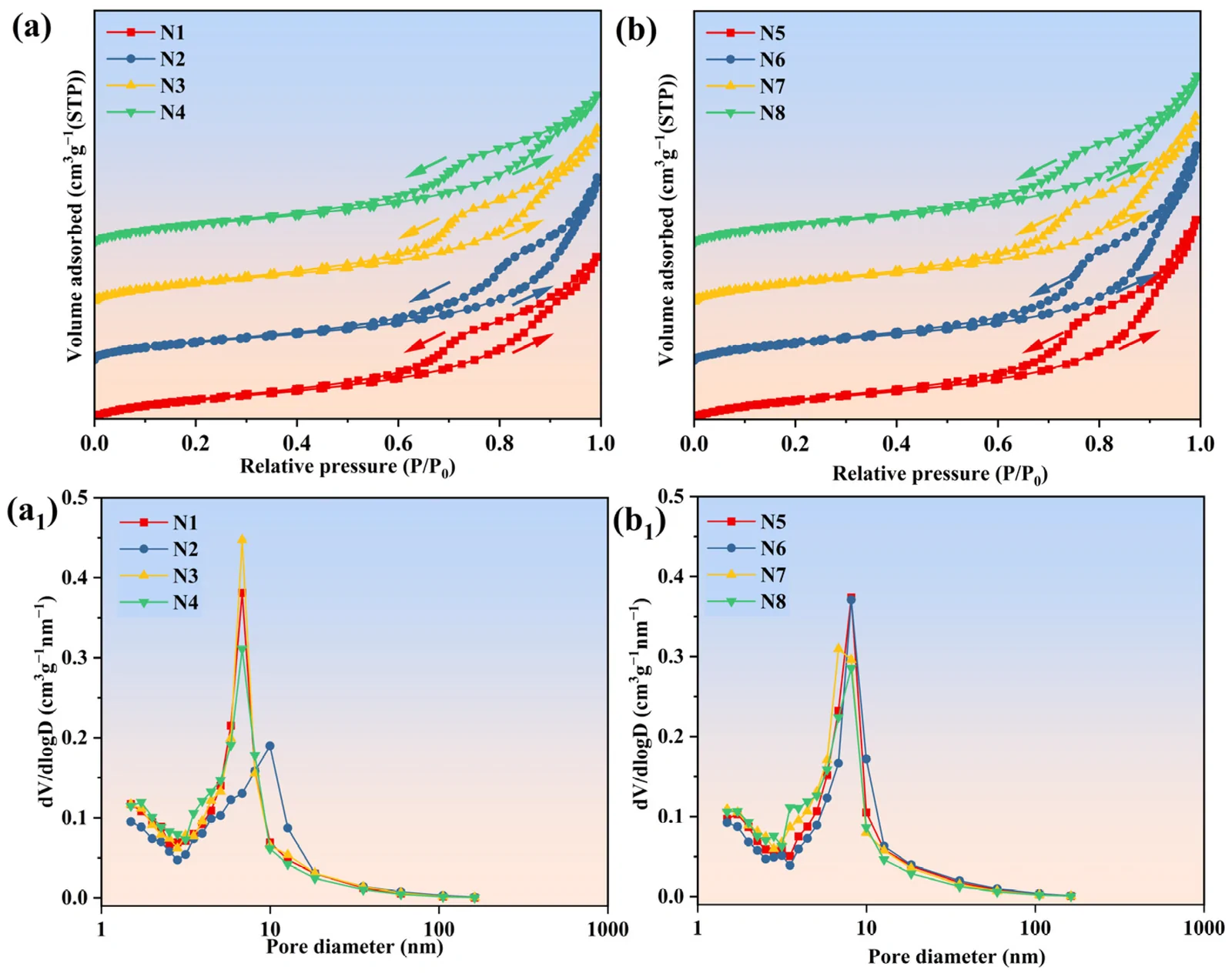
(**a**,**b**) N_2_ adsorption–desorption isotherm curves of synthesized samples; (**a_1_**,**b_1_**) pore size distribution curves of synthesized samples.

In terms of the specific surface area, the Barrett, Joyner and Halenda (BJH) adsorption pore diameters and total pore volumes of the samples are listed in Table 8 according to the above orthogonal experimental design. Pore structure and specific surface areas were measured and are shown in Figure 6.

As shown in Figure 6a,b, the SAs prepared from CTs all had the characteristics of type IV adsorption isotherms with H1 hysteresis loops [41]. As shown in Figure 6(a_1_,b_1_), the pore diameters of the eight samples were mainly between 2 and 20 nm. The specific surface areas were more than 700 m^2^/g for all studied samples, with N4 showing the highest surface area at 772.83 m^2^/g. The BJH adsorption pore diameters were in the range of 7.23 to 10.29 nm. The total pore volume was in the range of 2.18 to 2.79 cm^3^/g. The pore volume ratio was 92.59%. The combination of the calcination temperature and alkali-to-solid mass ratio had a substantial influence on the gelation time. Specifically, when the calcination temperature was 700 °C and the alkali-to-solid mass ratio was 0.75, the gelation time was more than 300 min, which is much longer than the other cases in Table 9. Generally, with the calcination temperature increase, the BJH adsorption pore diameters increased. When the alkali-to-solid mass ratio was kept constant at 0.875, the specific surface area was slightly higher than the case at 0.75.

Although sample N7 exhibited the highest specific surface area, sample N4 possessed the lowest bulk density at 0.136 g/cm^3^ under a calcination temperature of 700 °C. Consequently, the thermal conductivity of sample N4 was characterized, with a measured value of 0.03571 W/(m·K).

#### 3.3.2. Contact Angle Test of Hydrophobic Modified Aerogel

**Figure 7:**
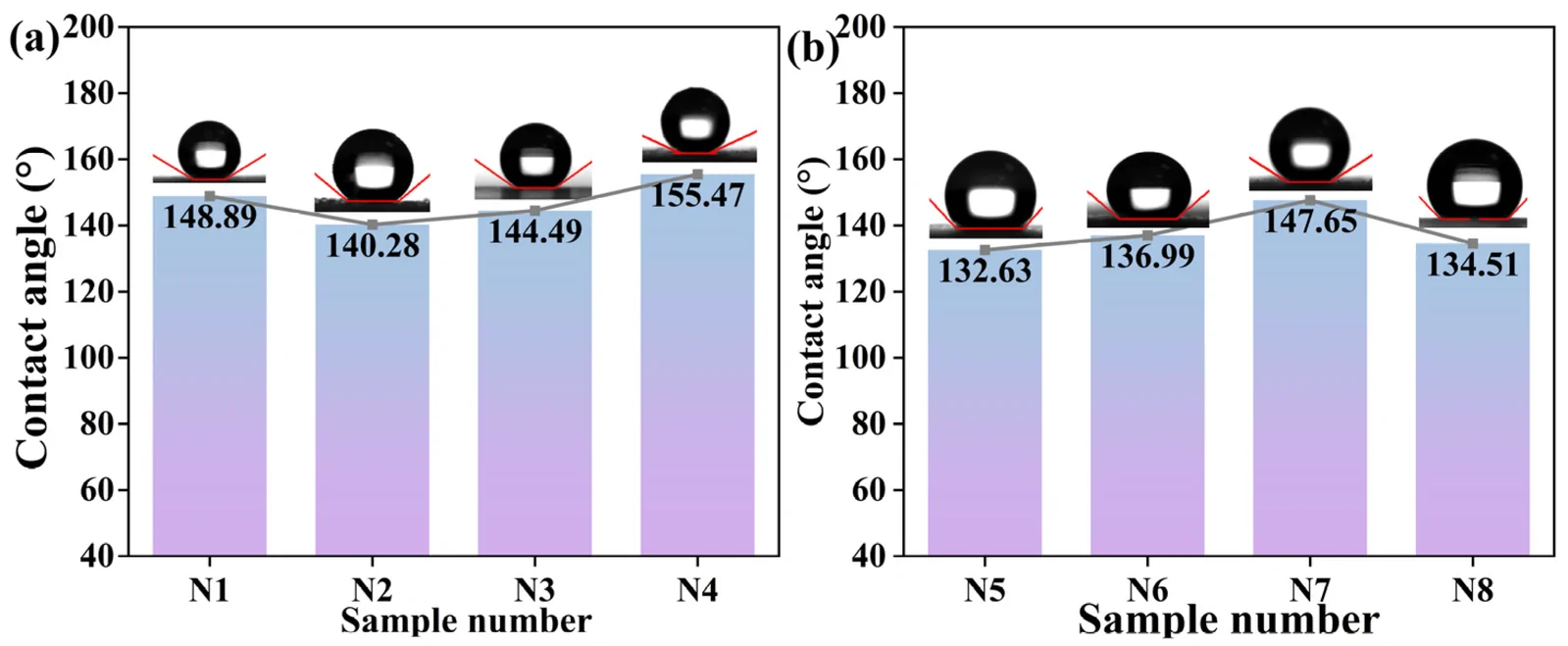
Contact angle of hydrophobically modified aerogel samples ((**a**) water contact angles of samples with different alkali-solid mass ratios and acid-solid mass ratios at an alkali fusion temperature of 700 °C; (**b**) water contact angles of samples with different alkali-solid mass ratios and acid-solid mass ratios at an alkali fusion temperature of 800 °C.).

After the formation of the wet gel, abundant hydrophilic groups were detected on its surface. During ambient pressure drying, the high surface tension of water readily induces the irreversible shrinkage of wet gel [42]. Therefore, hydrophobic modification of the wet gel before APD was beneficial to reduce the volume shrinkage and maintain the porous structure, which played an important role in stabilizing the properties at a later stage. The hydrophobic modification mechanism is shown in Equations (9) and (10) [43]. As shown in Figure 7, after hydrophobic modification by TMCS, all samples showed excellent hydrophobic properties (contact angles greater than 90°), and the hydrophobic angle of sample N4 was up to 155.47°:

(9) $${(CH}_{3}{)}_{3}-Cl+\equiv Si-OH\to \equiv {Si-O-Si(CH}_{3}{)}_{3}+HCl$$

(10) $${(CH}_{3}{)}_{3}Si-{OC}_{2}H_{5}+\equiv Si-OH\to \equiv {Si-O-Si(CH}_{3}{)}_{3}+{CH}_{3}{CH}_{2}OH$$

#### 3.3.3. Microstructure of Hydrophobically Modified Silica Aerogel

**Figure 8:**
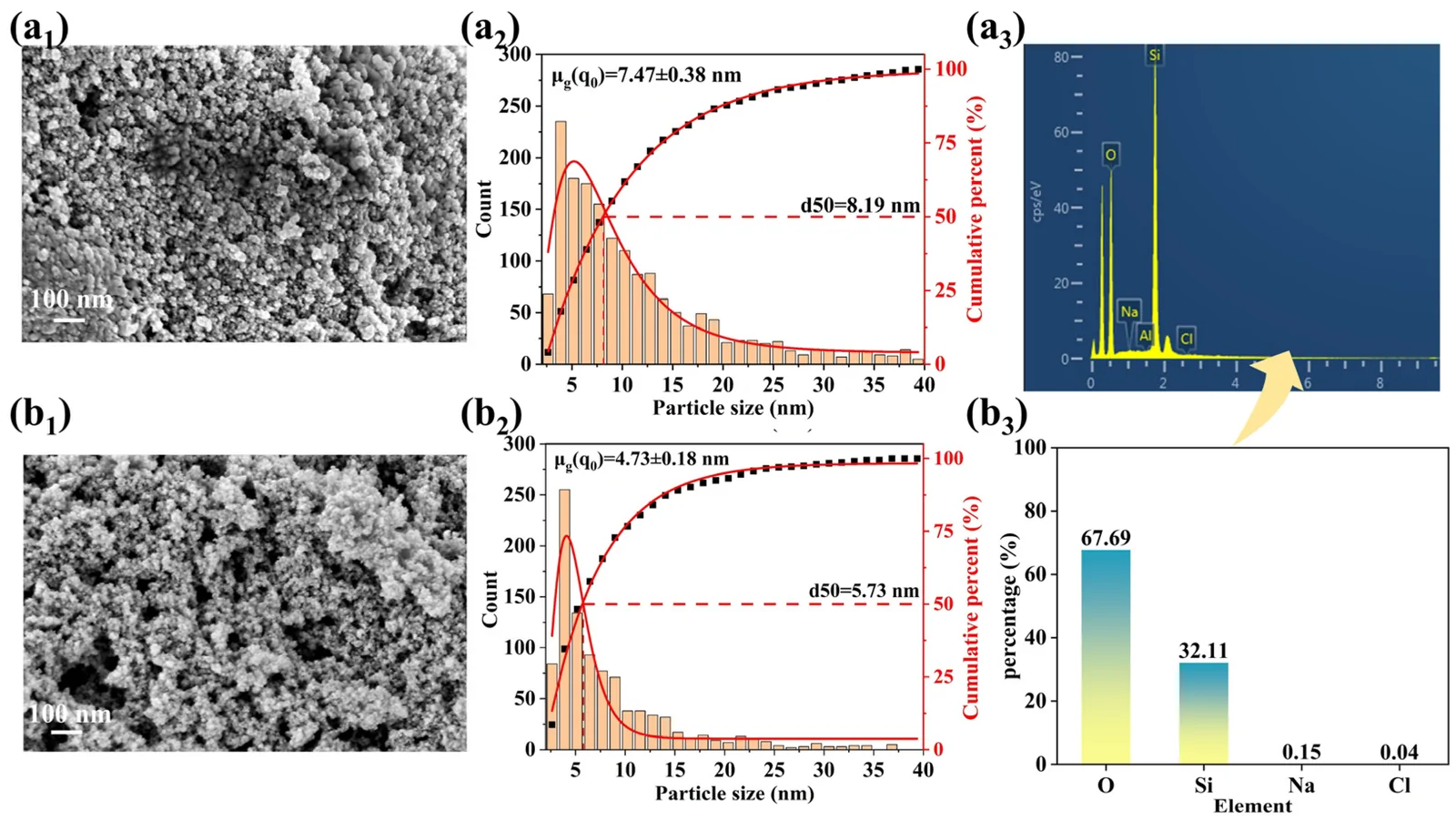
SEM images of samples N4 (**a_1_**) and N7 (**b_1_**); pore size distribution curves of samples N4 (**a_2_**) and N7 (**b_2_**); EDS analysis of sample N4 (**a_3_**,**b_3_**).

To better understand their microstructures, SEM images of samples N4 and N7 are presented in Figure 8. Generally, the silica aerogel was composited by small particles with median particle diameters less than 10 nm, and the pore structure was uniform. Image J software (ImageJ 1.54k, LOCI, Madison, WI, USA) was used to process the SEM images and analyze the size distribution of typical silica nanoparticles [44]. As shown in Figure 8(a_2_,b_2_), the silica aerogels of samples N4 and N7 had median diameters of 8.19 nm and 5.73 nm, respectively. Sample N4 had a smaller median diameter and larger BET specific surface area (as shown in Table 8) than sample N7. However, its BJH adsorption pore diameter is larger than that of N7. The acid-to-solid mass ratio had the most significant effect on the gelation time of the sol. Since the acid-to-solid mass ratio of N7 is smaller than that of N4, the strength of Si-O bonds during the sol–gel condensation process could have been lower, combined with the capillary pressure during APD, leading to the destruction of the three-dimensional network structure and an increase in the number of macropores (as shown in Figure 8(b_1_)). The EDS image in Figure 8(a_3_) shows the elementary composition of the silica gel. Extremely low Na^+^ and Cl^−^ contents were observed, indicating that through processes such as ultrasonic washing, solvent exchange and surface hydrophobic modification, most of the Na^+^ in the aerogel matrix was removed (as shown in Figure 8(b_3_)). The chemical composition analysis of the N4 sample shown in Table 9 confirms that the aerogel materials were mainly composed of silicon and oxygen, confirming the EDS results. A reasonable frequency range created cavitation bubbles in solvents and, with cavitation bubbles, implosion, energy released that served to provide micro-agitation at the water–contaminant silica wet gel interface. This micro-agitation enhanced the efficiency of the interaction between the solvent and the impurity ions being removed.

### 3.4. Thermal Stability Evaluation of Silica Aerogels

The thermal stability of the N4 sample was further investigated. TG-DSC was conducted on its weight loss. Heat treatment at different temperatures for 0.5 h was used to evaluate its thermal endurance. Hydrophobic angle, XRD and FTIR measurements were used for the characterization of the calcinated aerogels.

**Figure 9:**
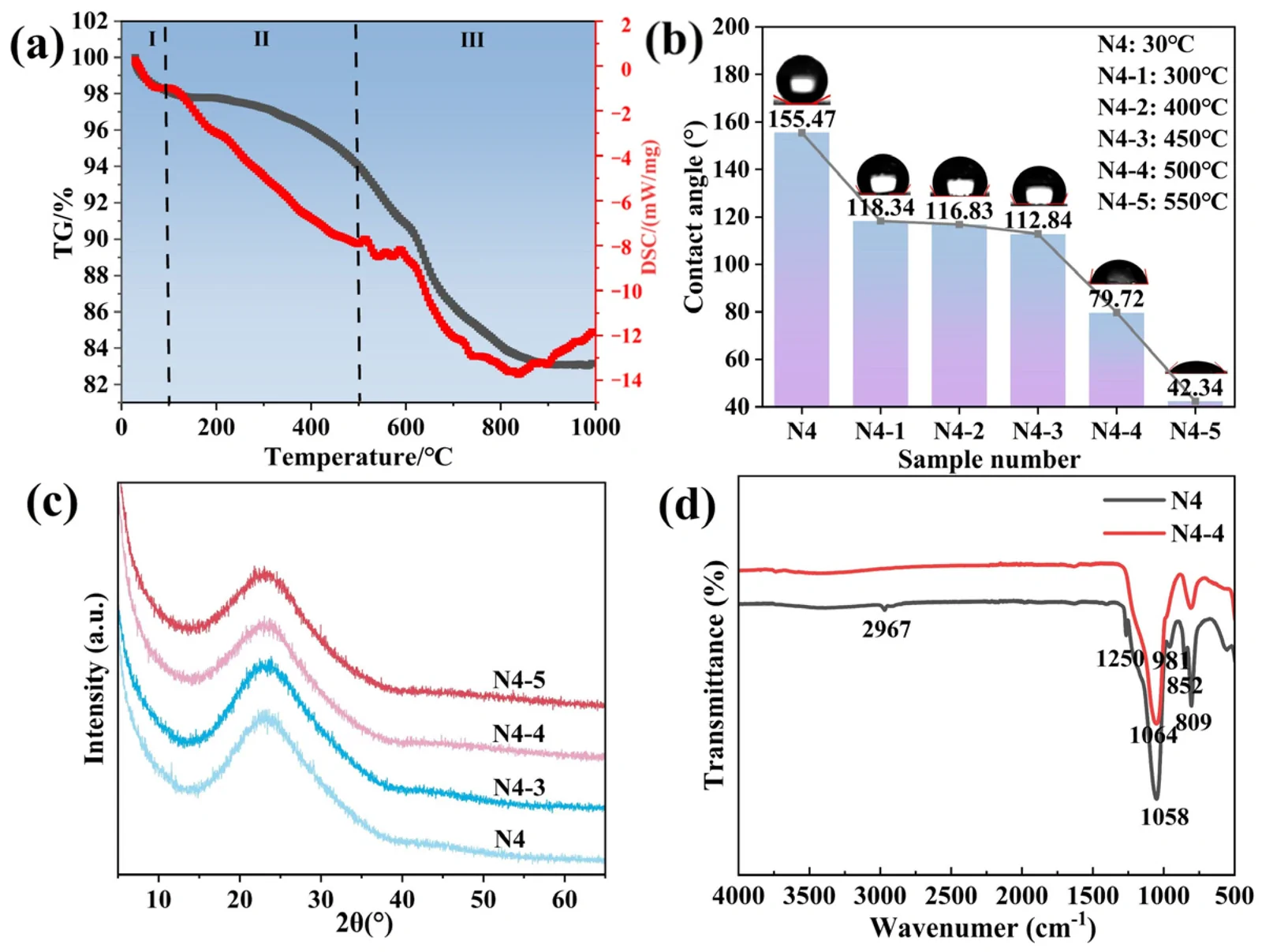
(**a**) TG-DSC curves of sample N4; (**b**) hydrophobic angle of sample N4 calcinated at different temperatures; (**c**) XRD pattern of sample N4 calcinated at different temperatures; (**d**) FTIR of N4 and N4-4 samples.

According to the TG-DSC results in Figure 9a, sample N4 exhibited a slow weight loss between 30 °C and 100 °C, which was attributed to the evaporation of water molecules. The mass loss between 100 °C and 500 °C was caused by the volatilization of small amounts of organic reagents, such as TMCS or hexane, on the aerogel surface [45]. An obvious endothermic peak appeared around 502 °C, which was due to the further decomposition of Si-CH_3_ groups on the silica aerogel during the hydrophobic modification process on the aerogel surface. The exothermic peak observed after 800 °C corresponds to the densification of the silica aerogel’s internal structure, where SiO_2_ underwent a crystallization process, transforming from an amorphous to a crystalline structure. The residual weight percentage of the SA aerogel reached 83.15 wt% at 1000 °C based on the TG results.

The hydrophobic angles of the calcinated samples are shown in Figure 9b. The hydrophobic angle at 300 °C decreased to 118.34° from 155.47° at 30 °C. After 450 °C, the hydrophobic angle decreased significantly and was reduced to 79.72° at 500 °C. The silica aerogel lost its hydrophobic properties when it calcinated at more than 500 °C. As the temperature increased, the Si-CH_3_ groups gradually decomposed, leading to a transition from hydrophobic to hydrophilic [21,46].

Based on the XRD results of sample N4 at different temperatures, there is only a wide peak between 10° and 30°, which was a typical feature of amorphous silica (as shown in Figure 9c). There is no significant crystalline diffraction peak. The broad peak of sample N4 between 10° and 30° gradually weakened with the increase in the calcination temperature, which could be attributed to the crystallization process of the aerogel during the calcination process.

The FTIR results of the N4 and N4-4 aerogel samples were analyzed and are shown in Figure 9d. The characteristic absorption peaks appearing near 809 cm^−1^ and 1058 cm^−1^ indicated the symmetric stretching vibration and antisymmetric stretching vibration of Si-O-Si bonds, respectively. The intensities of the above-mentioned peaks were significantly higher than those of other characteristic peaks, which demonstrated that this functional group had a relatively high content in the aerogels and served as the main component of the aerogel network structure. The characteristic peaks at 2967 cm^−1^, 1250 cm^−1^, 981 cm^−1^ and 852 cm^−1^ were assigned to the symmetric bending vibration peaks of the -CH_3_ groups, confirming that TMCS had successfully achieved the surface hydrophobic modification of the aerogels. When the calcination temperature increased to 500 °C, the hydrophobicity disappeared, with the -CH_3_ groups decomposed.

**Figure 10:**
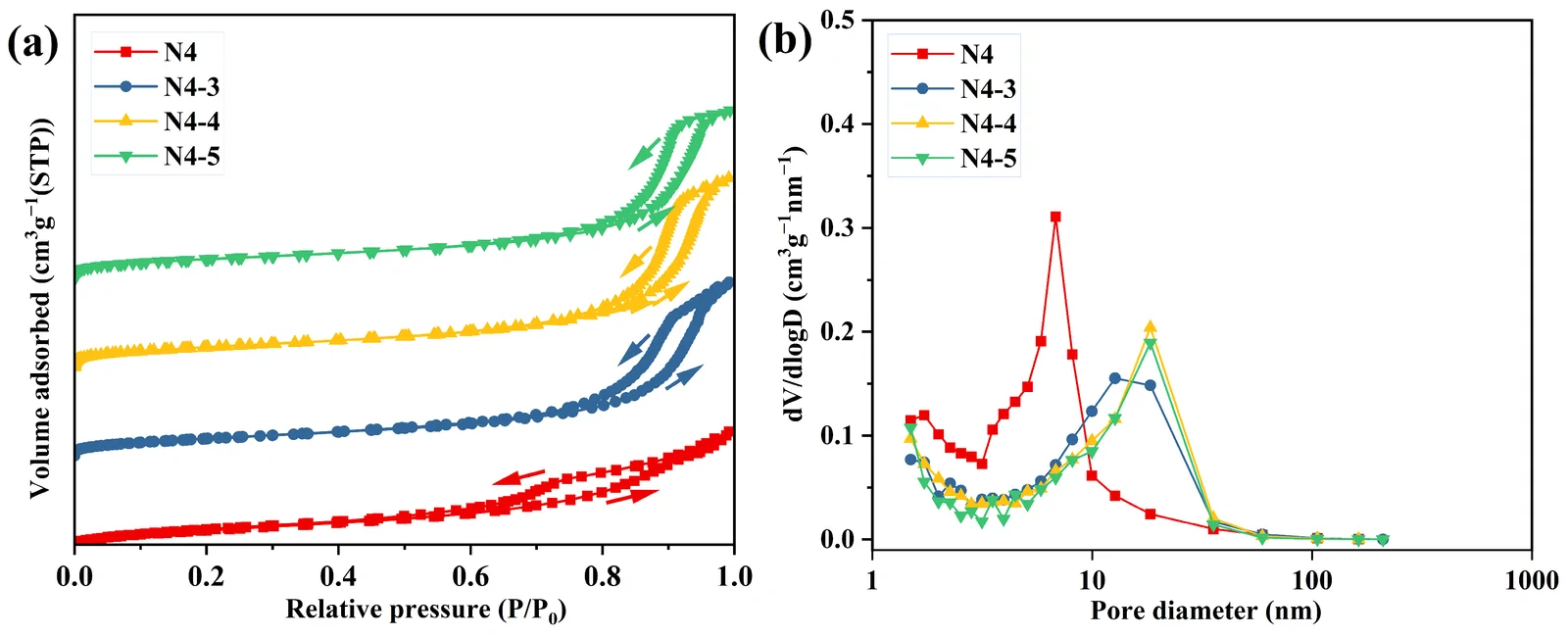
(**a**) N_2_ adsorption–desorption isotherm curves of sample N4 calcinated at different temperatures; (**b**) pore size distribution curves of sample N4 calcinated at different temperatures.

The BET specific surface areas and pore size distributions of samples calcined at different temperatures were analyzed and are shown in Table 10 and Figure 10. In Table 10, with the increase in the calcination temperature, the specific surface area of the aerogel gradually decreases while the pore volume and BJH adsorption pore diameters increase. Notably, after calcination, the BJH adsorption pore diameters of the samples were greater than 10 nm (shown in Figure 10). This can be attributed to the hydrophobic groups lost during the calcination at 500 °C. However, since the three-dimensional network structure of the aerogel material was still retained at this time, it still possessed a specific surface area of 603.19 m^2^/g at 550 °C and the typical characteristics of amorphous silica. The thermal conductivity at 450 °C with a specific surface area of 653.71 m^2^/g was 0.04286 W/(m·K).

### 3.5. Pore Structure Optimization Mechanism During APD

**Figure 11:**
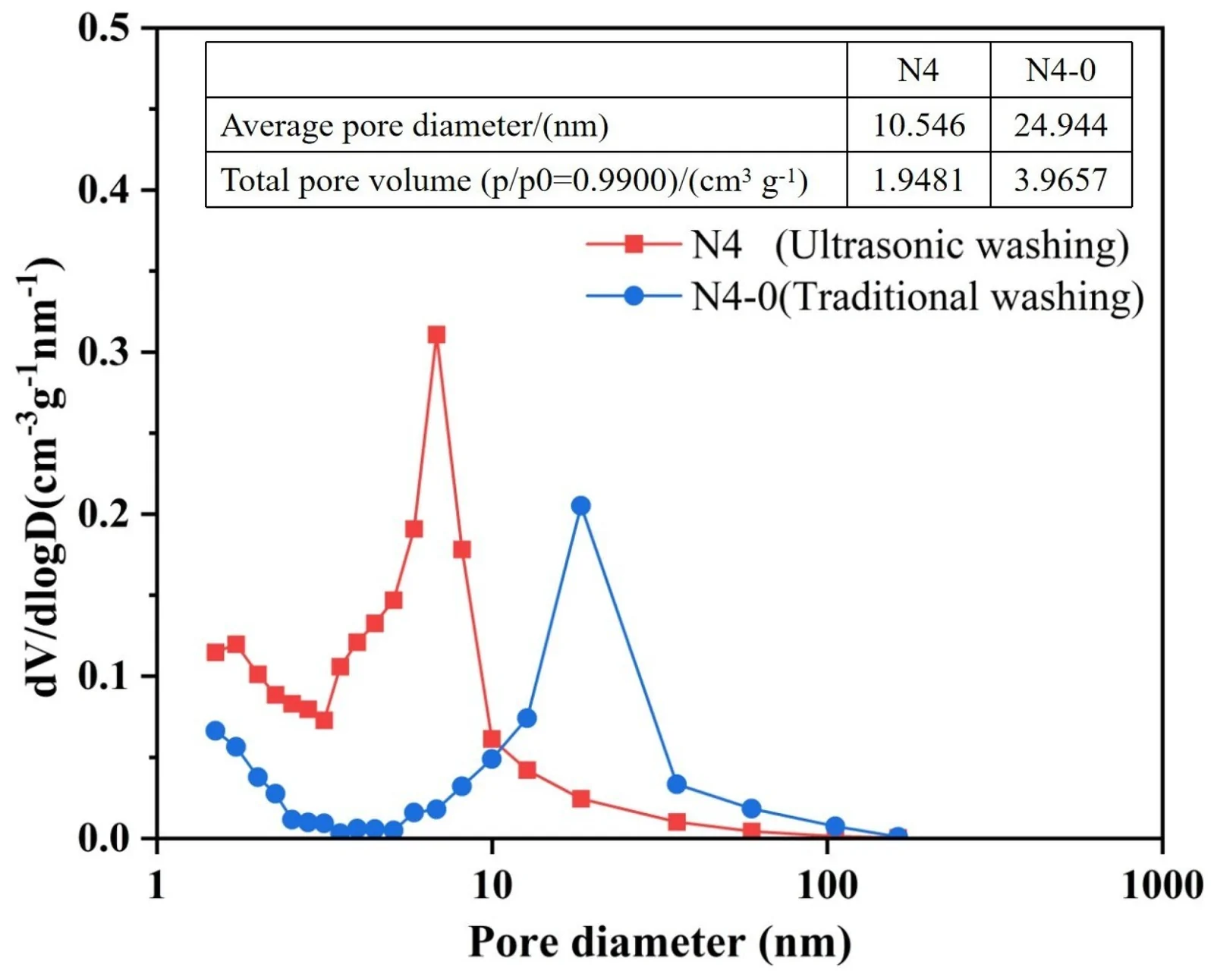
A comparison of the pore sizes and pore volumes of the N4 sample under traditional washing and ultrasonic washing under the same ratio.

The ambient pressure drying of aerogels is challenging due to the severe capillary pore-induced shrinkage of the nanoscale network. Firstly, we designed the intermittent ultrasonic washing step to remove cations. Through cavitation-induced micro-agitation, ultrasonic energy enhances the migration and extraction of residual cations (Na^+^, Mg^2+^, Ca^2+^) from the gel matrix. This resin-free purification strategy removes most of the impurity ions, significantly reducing capillary stress during APD and preserving the integrity of the pore structure. To reveal the advantages of intermittent ultrasonic washing, a control sample using conventional stirring washing without ultrasonic treatment was prepared (sample N4-0 in Figure 11). The comparison showed that intermittent ultrasonic washing decreased the hole collapse during ambient pressure drying. Secondly, hydrophobic modification using TMCS changed surface Si–OH to Si–CH_3_ groups, preventing hydrogen bonding and structural collapse during solvent evaporation. The resulting aerogels exhibited high specific surface areas (>700 m^2^/g), uniform mesoporous distributions (2–20 nm), and porosities exceeding 90%, endowing the aerogel with good thermal stability and low thermal conductivity.

The superior thermal insulation of the aerogel is primarily attributed to its low bulk density that suppresses thermal conduction, abundant nanopores that reduce thermal convection, and a homogeneous pore structure that weakens thermal radiation. Under the condition of the visual trend (calcination at 700 °C, an alkali-to-solid ratio of 0.875 and an acid-to-solid ratio of 10), sample N4 achieved a minimum bulk density of 0.145 g/cm^3^, and the fabricated aerogel still retained hydrophobicity at 502 °C, showing a better performance than many solid-waste-derived aerogels, as shown in Table 11.

## 4. Discussion

**Figure 12:**
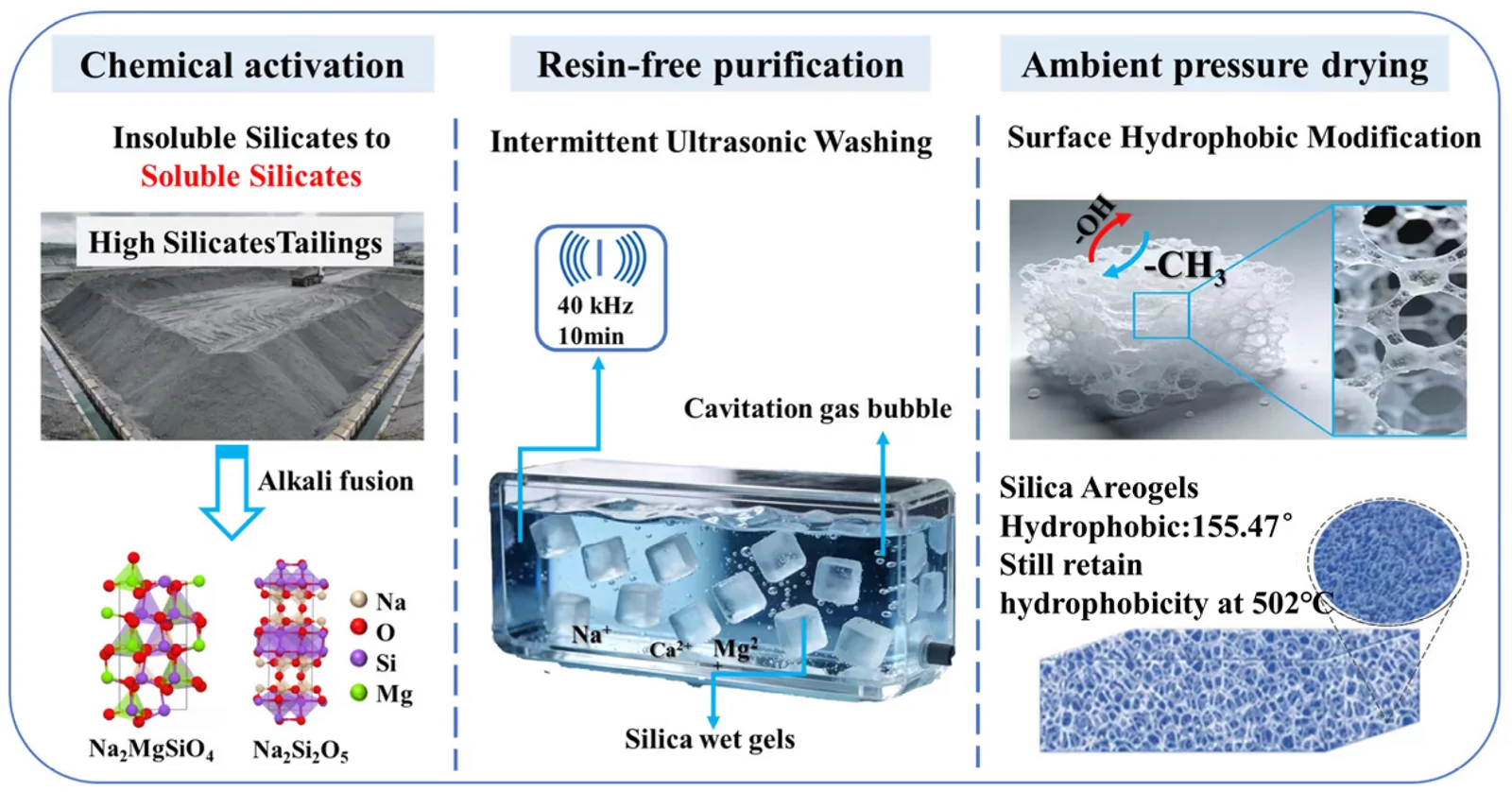
Schematic diagram of process mechanism and related characteristics of preparing silica aerogel materials from CTs.

The process mechanism and related characteristics of preparing silica aerogel materials from CTs can be further described by Figure 12. CTs are primarily composed of highly crystalline calcium and/or magnesium silicate minerals, such as olivine, enstatite, and amphibole. Their dense lattice structures and stable Si–O bonds render them poorly reactive under conventional acid leaching. Alkali fusion with NaOH effectively disrupts these silicate frameworks, converting them into soluble sodium silicates (e.g., Na_2_MgSiO_4_ and Na_2_Si_2_O_5_), thereby enhancing silicon availability for lateral hydrolyze.

During acid leaching, sodium silicates hydrolyze to form H_2_SiO_3_, as in Equations (11) and (12). Afterwards, the silica sol undergoes condensation under nitric acid catalysis to generate a three-dimensional SiO_2_·H_2_O gel network, as in Equation (13). A reasonable acid-to-solid ratio in this process has two effects: (1) neutralization of residual alkali in the alkali fusion process; (2) catalyzation of the polycondensation of silica sol to gel, thereby avoiding excessive protonation and facilitating the formation of a robust and homogeneous gel skeleton. Therefore, the design of the alkali-to-solid mass ratio and acid-to-solid mass ratio laid the foundation for the subsequent sol–gel transformation with a more homogeneous microstructure. Then, a resin-free purification strategy based on intermittent ultrasonic washing method was introduced to remove impurity ions and stabilize the gel network. After ultrasonic impurity removal, the wet gel was hydrophobically modified, which effectively solved the shrinkage problem during atmospheric drying:

(11) $${Na}_{2}{MgSiO}_{4}+4{HNO}_{3}\to {H_{2}SiO}_{3}+{Mg({NO}_{3})}_{2}{+2NaNO}_{3}+H_{2}O$$

(12) $${Na}_{2}{Si}_{2}O_{5}+2{HNO}_{3}+H_{2}O\to {{2H}_{2}SiO}_{3}{+2NaNO}_{3}$$

(13) $$H_{2}{SiO}_{3}\to {SiO}_{2}\cdot H_{2}O\left(amorphous\right)$$

## 5. Conclusions

The utilization of copper–nickel tailings as a silica source for aerogel synthesis aligns well with circular economy principles; however, the technology is still in the early stage of development. Its circular economic benefits can only be fully unlocked when two key challenges are addressed: improving the utilization efficiency of the tailings’ components and effectively controlling the formation of secondary wastes. This study demonstrates a feasible and promising strategy for converting copper–nickel tailings (CTs) into high-performance silica aerogels via alkali fusion activation, acid leaching and ambient pressure drying.

(1) The Box–Behnken experimental design method was employed to realize adjustable reaction degrees (up to 91.6%) of CTs and regulated gelation times (minimum 104 min) for silica sol. Alkali fusion transforms silicate minerals in CTs into soluble sodium silicates, according to the XRD results, while nitric acid eliminates residual NaOH and accelerates the polycondensation of wet gels, which provides robust theoretical support for improving SiO_2_ usage in CTs.

(2) The intermittent ultrasonic washing treatment enabled the removal of the vast majority of impurity Na^+^ and effectively inhibited the volume shrinkage of aerogels during ambient pressure drying.

(3) The prepared SA aerogel delivers an excellent comprehensive performance, including a high specific surface area of 772.83 m^2^/g, a low bulk density of 0.145 g/cm^3^, and a low thermal conductivity of 0.03571 W/(m·K). The fabricated aerogel can still retain a certain hydrophobicity at 502 °C.

## Figures and Tables

**Table 1:** Main chemical compositions of CTs (wt.%) determined from XRF.

Oxides	SiO_2_	MgO	Fe_2_O_3_	Al_2_O_3_	CaO	S	Na_2_O	TiO_2_	Cr_2_O_3_	NiO	CuO	LOI
CTs	40.40	27.06	13.74	6.70	4.20	1.40	0.79	0.32	0.26	0.14	0.05	4.33

**Table 2:** Chemical reagents used in experimental process.

Reagent Name	Purity	Manufacture
EtOH	AR, ≥99.7%	Wuxi Ya Sheng Chemical Co., Ltd. Wuxi, China.
Deionized water	/	Nanjing Wan Qing Chemical Glassware Instrument Co, Ltd. Nanjing, China.
N-hexane	AR, ≥97%	Sinopharm Chemical Reagent Co., Ltd. Shanghai, China.
Nitric acid	GR, ≥97.2%	Sinopharm Chemical Reagent Co., Ltd. Shanghai, China.
Trimethylchlorosilane	GC, ≥98.0%	Shanghai Aladdin Biochemical Technology Co., Ltd. Shanghai, China.

**Table 3:** The rules abbreviations used in this work.

Abbreviations	Full Name
CTs	Copper–nickel tailings
BBD	Box–Behnken design
SA	Silica aerogel
APD	Ambient pressure drying
TMCS	Trimethylchlorosilane
XRD	X-ray diffraction
XRF	X-ray fluorescence
EDS	Energy-dispersive X-ray spectroscopy
TG-DSC	Thermogravimetry–differential scanning calorimetry
FTIR	Fourier transform infrared spectroscopy
SEM	Scanning electron microscope

**Table 4:** Box–Behnken response surface experimental design.

Factor	Level		
	Low Level (−1)	Medium Level (0)	High Level (1)
Calcination temperature (A)	600	700	800
Alkali-to-solid mass ratio (B)	0.5	0.75	1
Acid-to-solid mass ratio (C)	6	8	10

**Table 5:** Box–Behnken response surface experiment table with obtained experimental results of reaction degrees of CTs and gelation times of silica sol.

Standard Order	Factor			Reaction Degree (%)	Gelation Time (min)
	A	B	C	Y1	Y2
S1	−1	−1	0	58.00	309
S2	1	−1	0	72.67	168
S3	−1	1	0	55.20	979
S4	1	1	0	81.60	610
S5	−1	0	−1	60.07	2181
S6	1	0	−1	69.34	633
S7	−1	0	1	70.14	268
S8	1	0	1	85.59	126
S9	0	−1	−1	72.39	133
S10	0	1	−1	77.02	1440
S11	0	−1	1	71.76	104
S12	0	1	1	79.52	427
S13	0	0	0	76.88	379
S14	0	0	0	76.00	360
S15	0	0	0	77.00	369
S16	0	0	0	76.50	380
S17	0	0	0	75.90	376

**Table 6:** Analysis of variance of reaction degree (Y_1_).

Source	Sum of Squares	Degree of Freedom	F Value	*p*-Value	Significance
Model	965.356	9	6.584	0.0106	Significant
A	541.041	1	33.212	0.0007	Very Significant
B	42.874	1	2.632	0.1488	Not Significant
C	99.335	1	6.098	0.0429	Significant
AB	34.398	1	2.112	0.1895	Not Significant
AC	9.548	1	0.586	0.46889	Significant
BC	2.499	1	0.150	0.7097	Not Significant
A^2^	191.161	1	11.734	0.0111	Significant
B2	34.212	1	2.100	0.1906	Not Significant
C2	10.339	1	0.635	0.4518	Not Significant
Residual	114.034	7			
Lack of Fit	113.039	3	151.519	0.0001	Very significant
R^2^ = 0.8947		Adj R^2^ = 0.7593		C.V. = 3.56%	

Note: significant (*p* < 0.05); very significant (*p* < 0.01); extremely significant (*p* < 0.0001).

**Table 7:** ANOVA of Ln Y_2_.

Source	Sum of Squares	Degree of Freedom	F Value	*p*-Value	Significance
Model	10.108	9	8.137	0.0057	Very significant
A	1.181	1	8.599	0.0222	Significant
B	4.863	1	35.232	0.0006	Very significant
C	3.344	1	24.299	0.0017	Very significant
AB	0.00	1	0.03	0.8596	Not Significant
AC	0.058	1	0.421	0.5369	Not Significant
BC	0.235	1	1.703	0.2331	Not Significant
A2	0.310	1	2.26	0.1768	Not Significant
B2	0.100	1	0.72	0.4248	Not Significant
C^2^	0.011	1	0.081	0.7846	Not Significant
Residual	0.966	7			
Lack of Fit	0.964	3	631.231	<0.0001	Significant
R^2^ = 0.9126		Adj R^2^ = 0.8002		C.V. = 4.01%	

Note: significant (*p* < 0.05); very significant (*p* < 0.01); extremely significant (*p* < 0.0001).

**Table 8:** Pore structure characteristics of aerogel samples.

Sample	N1	N2	N3	N4	N5	N6	N7	N8
Calcination temperature (°C)	700	700	700	700	800	800	800	800
Alkali-to-solid mass ratio	0.75	0.75	0.875	0.875	0.75	0.75	0.875	0.875
Acid-to-solid mass ratio	8	10	8	10	8	10	8	10
Specific surface area (m^2^/g)	732.39	704.32	744.80	772.83	742.01	707.41	738.92	745.49
BJH adsorption pore diameter (nm)	7.74	9.69	7.99	8.42	9.27	10.29	7.23	8.11
Total pore volume (cm^3^/g)	2.51	2.35	2.26	2.47	2.64	2.79	2.72	2.81
Bulk density (g/cm^3^)	0.207	0.178	0.179	0.145	0.160	0.168	0.136	0.172
Reaction degree (%)	76.46	76.68	74.06	91.60	74.32	69.34	85.59	80.63
Gelation time (minute)	373	302	379	126	168	132	129	149

**Table 9:** XRF analysis of N4 aerogel powder samples.

Oxides	SiO_2_	Cl	Na_2_O	MgO	Fe_2_O_3_	SO_3_	TiO_2_	CaO	Cr_2_O_3_	NiO	LOI
N4	99.05	0.43	0.27	0.06	0.05	0.04	0.04	0.03	0.002	0.001	0.027

**Table 10:** Pore structure characteristics of sample N4 calcinated at different temperatures.

Number	Specific Surface Area (m^2^/g)	BJH Adsorption Pore Diameter (nm)	Total Pore Volume (cm^3^/g)
N4	772.83	8.42	2.47
N4-3	653.71	12.37	3.03
N4-4	624.23	13.02	3.31
N4-5	603.19	12.89	2.96

**Table 11:** Comparison of microstructure and thermal properties of aerogel materials prepared from solid waste by APD method.

Silicon Source	Bulk Density (g/cm^3^)	S_BET_ (m^2^/g)	Decomposition Temperature of Surface Si-CH_3_ Hydrophobic Groups (°C)	Ref.
Rice husk ash	0.071	649.10	400	[45]
Kaolin	0.150	465.30	/	[47]
Fly ash	0.085	809.92	400	[48]
Coal gangue	/	748.00	476	[46]
Coal gangue	0.230	416.00	/	[30]
Iron tailings	/	683.84	/	[49]
CTs	0.145	772.83	502	This work

## Data Availability

The original contributions presented in this study are included in the article. Further inquiries can be directed to the corresponding authors.
